# Supplementary material for: Content-rich biological network constructed by mining PubMed abstracts
Source: BMC Bioinformatics. 2004 Oct 8;5:147. doi: 10.1186/1471-2105-5-147 (PMC528731; doi:10.1186/1471-2105-5-147)
Supplement: Additional File 5 — The original Chilibot query results of the term "long-term potentiation (LTP)" and 22 other terms, limiting the latest references analyzed to the years 1990, 1995, 2000, and 2004. [file 1471-2105-5-147-S5.bz2 › chilibotAdditionalFile5/ltp2004/html/ARC_SYNAPTOPHYSIN.html]

 


 **ARC** and **SYNAPTOPHYSIN** 
  
Found 6 abstracts in PubMed,  **6 abstracts were retrieved and analyzed**.  


---

 Search Google  |
 PDF files only 
|  EDU domain only 

---

**Interactive relationship** (e.g. stimulation, inhibition, etc)

- Immunoblot analyses of the POA and ME  **ARC**  region of the hypothalamus revealed a significant increase in the content of PSA NCAM, NCAM 180, NCAM 140, GAP 43 and  **synaptophysin**  from proestrous phase rats as compared to diestrous phase rats.  Ref: 12393229 Brain Res, 2002

- :-)

  **Parallel relationship** (e.g. studied together, co-existance, homology, etc.)

  - 2 Research on neural plasticity related sensitization revealed the involvement of several molecules such as tissue plasminogen activator,  **arc** ,  **synaptophysin**  and stathmin.  Ref: 11233297 Nippon Yakurigaku Zasshi, 2001
  - The B16 labelling pattern in the retina was compared with that of two synaptic vesicle antigens SV2 and anti  **synaptophysin**  in the outer plexiform layer were more diffuse and apparently filled the entire presynaptic terminal whereas B16 labelling was more restricted and labelled a discrete structure resembling a semi ellipse or an  **arc**  with the ends pointing to the inner nuclear layer and the middle curve facing the outer nuclear layer 1 2 microns long by <.  Ref: 8971636 J Neurocytol, 1996
